# Leukotriene receptor antagonists for chronic urticaria: a systematic review

**DOI:** 10.1186/1710-1492-10-24

**Published:** 2014-05-07

**Authors:** Nipun Lakshitha de Silva, Hasitha Damayanthi, Anoja Chamarie Rajapakse, Chaturaka Rodrigo, Senaka Rajapakse

**Affiliations:** 1 Department of Clinical Medicine, Faculty of Medicine, University of Colombo, 25 Kynsey Road, Colombo 08, Sri Lanka; 2 Department of Pharmacology, Faculty of Medicine, Colombo, Sri Lanka; 3 Department of Geriatrics, Kings Mill Hospital, Sherwood Forest NHS Foundation Trust, Sutton-in-Ashfield, Nottinghamshire, UK

**Keywords:** Chronic urticaria, Leukotreine receptor antagonists, Leukotreine, Montelukast, Zafirlukast, Antihistamines

## Abstract

A significant proportion of patients with chronic urticaria respond inadequately to first line treatment with antihistamines. Leukotreine receptor antagonists (LTRA) are also used for chronic urticaria, although firm recommendations on their use are lacking. We performed a systematic review of randomised trials to determine the role of LTRA in treatment of chronic urticaria. A search of PUBMED, EMBASE, SCOPUS, LILACS, the Cochrane Central Register of Controlled Trials, and the Web of Science for relevant randomized control trials or cross over studies yielded 10 eligible studies. The heterogeneity of trials were high, preventing valid meta-analysis of data. Most trials indicated that LTRA are not superior to placebo or antihistamine therapy, while combination therapy of LTRA and antihistamines appear to be more efficacious compared to antihistamine alone. The side effect profile and tolerability of this group of drugs is acceptable. The use of LTRA as monotherapy cannot be recommended. LTRA are effective add-on therapy to anti-histamines, and their use in patients responding poorly to antihistamines is justifiable. Further well designed randomized controlled trials with clear and standardized outcome measures are needed to determine the role of LTRA in chronic urticaria.

## Introduction

Urticaria (hives) is a condition characterized by superficial skin weals and pruritus. Classically, chronic urticaria is defined as the daily, or almost daily, occurrence of urticarial weals for at least 6 weeks, without any physical, allergic, infectious, drug-related or vasculitic cause [1,2]. In the past, chronic urticaria was divided into two types, i.e., chronic autoimmune urticaria and chronic idiopathic urticaria [3]. The current classification defines these as chronic spontaneous urticaria (CSP), characterised by the occurrence of spontaneous weals and/or angio-oedema for longer than 6 weeks [4]. Several chemical mediators such as histamines, leukotrienes, prostaglandins and cytokines secreted by mast cells and basophils are involved in the pathogenesis of urticaria [5]. However, the routine treatment of autoimmune and non-autoimmune chronic urticaria is similar [1,2,6].

A step-wise approach to treatment is currently advocated in the EAACI/GA^2^LEN/EDF/WAO 2009 treatment guidelines [7]. First line therapy is with a non-sedating H_1_-antihistamine at standard doses. If no response is seen after two weeks, the dose is increased up to four times the standard or licensed dose. The addition of a leukotriene receptor antagonist (LTRA) is recommended as third line treatment. For severe or resistant cases, immunosuppressants such as ciclosporin, dapsone, H_2_-antihistamines and omalizumab [8] are also used. Short-course systemic steroids are recommended for exacerbations.

Cysteinyl leukotrienes are potent pro-inflammatory mediators whose effect can be blocked by LTRA such as montelukast, zafirlukast and pranlukast. The benefit of these drugs in patients suffering from asthma and allergic rhinitis is well established, and current evidence clearly justifies their use in those conditions [9]. Several recent studies have addressed the use of these agents, as monotherapy as well as in combination with other first line therapies, for chronic urticaria. Nonetheless, there is controversy as to the efficacy of LTRA for this indication; a previous review of six randomised clinical trials comparing the use of montelukast either alone or in combination with non-sedating antihistamines concluded that the presence of contradictory results makes the recommendation of montelukast for treating chronic urticaria not justifiable [10]. Another systematic review on the subject which included several case-series, open-labelled studies and randomised controlled trials also concluded that LTRA are not beneficial alone or in combination with antihistamines in patients with idiopathic chronic urticaria, although benefit was seen in patients with food additive hypersensitivity and NSAID-exacerbated chronic urticaria [11].

Evidence from new trials have been published since then. In this context it is timely to evaluate the evidence from randomized control trials on this subject, in order to provide an answer to the clinical question as to whether prescribing LTRAs for chronic urticaria is justifiable. Hence, we performed a systematic review of randomized controlled trials examining the efficacy of LTRA either alone or in combination with other drugs, compared with placebo or antihistamines, for the treatment of chronic urticaria.

## Review

### Methods

Our aim was to include randomised controlled trials (including cross-over studies) on patients with chronic urticaria treated with LTRA alone or in combination with antihistamines, compared with placebo or antihistamines alone. We did not include physical urticaria as this is thought to be pathophysiologically different [2]. We searched MEDLINE, EMBASE, LILACS, Cochrane Central Register of Controlled Trials (CENTRAL), Web of Science and SCOPUS SciVerse for English language publications, using the search terms ‘montelukast’ OR ‘*lukast’ OR ‘zafirlukast’ OR ‘leukotriene’ AND ‘urticaria’ in title, abstract, keywords or topic with no date limits. The reference lists were imported into ENDNOTE X5®, and duplicates were removed. Two reviewers independently evaluated the abstracts of all retrieved publications and selected studies likely to provide relevant data. Full texts of selected publications were retrieved, and the two reviewers independently read through the full texts. Mean difference was used as the effect measure and 95% confidence interval was taken as the level of significance.

Our search hits were as follows: combined search of MEDLINE and EMBASE = 103, Web of Science = 220, Scopus SciVerse = 846, CENTRAL = 31, LILACS = 217. After filtering, we identified 13 randomised controlled trials (RCT) which provided relevant data, and all were included in the systematic review. Two studies evaluated the role of LTRA in delayed pressure urticaria, and were excluded. Another study which evaluated the role of LTRA in food additive hypersensitivity was excluded, thus leaving 10 trials for the final analysis. We obtained the full texts in 9, and the other was published only in abstract form [12].

### Results

Selected studies had comparisons of LTRA *vs*. placebo, LTRA *vs*. antihistamines, and combination therapy (LTRA plus antihistamines) *vs*. antihistamines alone. There was much variation in the outcome measures studied; these included different types of scores, marked either by the investigator or the patient, pertaining to clinical improvement of urticaria, pruritus, number of hives, size of largest hives, effect on daily activities and sleep. Total Symptom Score (TSS) [13,14], Urticarial Activity Score (UAS) [15], use of rescue medication and the visual analogue scale were considered as outcomes of overall improvement (Table 1). Tolerability and safety of the treatments were assessed based on symptomatology and changes in laboratory parameters. The heterogeneity of the studies was too high to perform pooling of data and valid meta-analysis. There were no studies evaluating the role of LTRA in angio-oedema per se.

**Table 1:** Summary of effects of LTRA in all studies

**Outcome measure**	**Studies**	**LTRA vs Placebo**	**LTRA + AH vs AH**	**LTRA vs AH**
Pruritus	Di Lorenzo et al.	ND	ND	Favours AH
	Reimers et al.	ND	-	-
	Kosnik et al.	-	ND	-
	Nettis et al. [14]	-	Favours LTRA	-
	Godse	-	-	Favours AH
Number of weals	Di Lorenzo et al.	Favours LTRA	ND	Favours AH
	Reimers et al.	ND	-	-
	Kosnik et al.	-	ND	-
	Nettis et al. [14]	-	Favours LTRA	-
Interference with sleep	Di Lorenzo et al.	ND	ND	Favours AH
	Reimers et al.	ND	-	-
	Kosnik et al.	-	ND	-
Interference with daily activities	Di Lorenzo et al.	Favours LTRA	ND	ND
	Reimers et al.	ND	-	-
Quality of life score	Agcaoili et al.	ND	-	-
	Nettis et al. [14]	-	Favours LTRA	-
TSS	Di Lorenzo et al.	Favours LTRA	ND	Favours AH
	Nettis et al. [23]	-	-	Favours LTRA
Size of weals	Di Lorenzo et al.	ND	ND	-
UAS	Erbagci et al.	Favours LTRA	-	-
	Agcaoili et al.	Favours LTRA	-	-
	Kosnik et al.	-	ND	-
	Wan et al.	-	ND	-
	Godse	-	-	Favours AH
Weekly AH count	Erbagci et al.	Favours LTRA	-	-
Visual analog scale	Bagenstose et al.	-	Favours LTRA	-
	Nettis et al.	-	Favours LTRA	-
	Wan et al.	-	ND	-
TES	Bagenstose et al.	-	ND	-

ND- no difference, AH- antihistamine, LTRA- leukotriene receptor antagonist, TSS – total symptom score, UAS- urticarial activity score, TES – treatment effectiveness score.

### LTRA vs. placebo

Di Lorenzo et al. [13] conducted a well-designed RCT of 160 adult (age 18–69 years) patients with chronic idiopathic urticaria, comparing four parallel groups receiving desloratadine 5 mg daily, desloratadine 5 mg daily plus montelukast 10 mg daily, montelukast 10 mg daily, and placebo. Patients with drug or food induced urticaria, physical urticaria, and urticaria-vasculitis were excluded. Treatment was given for 6 weeks, with assessment at 3 and 6 weeks and 2 weeks after completing treatment. Symptoms were scored daily, using reflective and instantaneous assessment. Compliance was better in patients randomized to desloratadine. Based on instantaneous assessment, montelukast was superior to placebo with regards to several outcome measures, such as TSS (p = 0.005) and number of hives (p = 0.001), although pruritus and size of largest hives did not show significant differences. Based on reflective evaluation, there was no difference in interference with sleep and the use of rescue medication (oral loratidine) with montelukast, however benefit was shown with regards to interference with daily activities (p = 0.002). All side effects reported were mild.

Erbagci et al. [16] compared montelukast 10 mg daily *vs.* placebo in a single-blind placebo-controlled cross-over trial (n = 30). In this study, patients used the antihistamine cetirizine on an ‘as required’ basis. Each treatment option was given for 6 weeks with a 2 week washout period. All patients completed the study. There was significant improvement in the UAS and weekly antihistamine count in the montelukast group. No significant side effects were reported attributable to the use of montelukast, and the tolerability profile was the same as for placebo. Five female patients developed transitory and mild headache.

A randomised double-blind placebo-controlled cross-over study was conducted by Riemers et al. [17] comparing zafirlukast 20 mg daily with placebo (n = 52). None of the outcome measures assessed either by the patient (itching score, number of weals score, disturbance of sleep score, disturbance of daily activity score and need of rescue medication score) or by the physician (intensity of erythema, number of weals, extension of weals, and size of weals) showed statistically significant differences in either group. No significant benefit was seen with zafirlukast even when the subset of patients with symptoms aggravated by pressure, NSAIDs or aspirin was considered. This is in contrast to other studies, where LTRA showed evidence of benefit in direct-pressure urticaria, [18] and food additive or aspirin induced urticaria [19]. Nonetheless, treatment with zafirlukast was well tolerated, with no clinical side effects or significant changes in laboratory parameters.

Agcaoili et al. [12] in a randomised double blind study published only in abstract form, compared montelukast to placebo in 29 patients with chronic urticaria. Outcome measures were difference in the UAS and mean quality of life score from baseline, on the second and fourth week of treatment; overall outcome was defined as success or failure rates. UAS was significantly improved after two weeks, and the quality of life score showed a trend towards improvement. Success rates were better with montelukast after two weeks.

### LTRA plus antihistamine vs. antihistamine alone

Bagenstose et al. [20] conducted a double-blind placebo-controlled trial to assess the benefit of add-on LTRA, by comparing cetrizine 10 mg daily plus zafirlukast 20 mg twice a day *vs*. cetirizine 10 mg daily plus placebo, for three weeks (n = 86). The study recruited only patients with sub-optimal response to antihistamine therapy. Patient and physician rated treatment efficacy using the Treatment Effectiveness Score (TES), and number and size of lesions by visual analogue scale (VAS) were evaluated at each weekly visit for 3 weeks. VAS scores assessed by both patients and physicians were significantly lower in the cetrizine plus LTRA group. There were no significant adverse effects or change in laboratory results in either groups.

The study by Di Lorenzo also compared combination therapy *vs*. anti-histamine monotherapy as mentioned above; combination therapy did not offer significant benefit over monotherapy with desloratadine.

A cross-over double-blind study of 24 patients with chronic urticaria whose symptoms were not adequately alleviated by antihistamines was conducted by Kosnik et al. [15] comparing montelukast 10 mg daily *vs.* placebo, in addition to the patients’ usual antihistamine therapy. Each treatment arm was continued for 2 weeks with a 7 day washout period in between. There was no significant difference in total symptoms and medication scores between the periods of placebo and montelukast use. A sub-group analysis of 5 patients with severe symptoms showed benefit with montelukast when an in-house severity scale (calculated by summing up the pruritus score, number of weals score and sleep disturbance score) was used; this benefit was not demonstrated when a validated UAS was used.

A randomised double-blind placebo controlled study was conducted by Nettis et al. [14] involving 81 patients, of whom 5 discontinued treatment, resulting in an evaluable population of 76. Patients were randomized to receive desloratadine 5 mg daily plus placebo, desloratadine 5 mg daily plus montelukast 10 mg daily, or placebo only for six weeks. This study demonstrated greater benefit with the combination of desloratadine plus montelukast compared to desloratadine alone in improving pruritus, number and size of weals, quality of life, and VAS. Improvement in the number of separate urticarial episodes did not show a significant difference between the two groups. There were no significant clinical, biochemical or electrocardiographic adverse effects seen with LTRA use. The study was well conducted and risk of bias was low, with clear details of blinding and minimal loss to follow-up.

Wan et al. [21] performed a single blind randomised controlled trial of 120 patients with newly diagnosed chronic urticaria. After a 1 week washout period, patients were randomised to four groups: oral hydroxyzine 25 mg + cetirizine 5 mg twice a day, oral hydroxyzine 25 mg + famotidine 20 mg twice a day, oral hydroxyzine 25 mg twice a day + montelukast 5 mg twice a daily, and oral placebo twice a day. The drop out rate among those receiving placebo was high, as the patients did not experience benefit (13 out of 30). UAS and a visual analog scale were used to evaluate outcome. The efficacy of the combination of antihistamine and LTRA was comparable with treatment with dual antihistamine therapy, and superior to placebo. The main difficulty in interpreting this study from a clinical perspective is that the comparison was with dual antihistamine therapy.

### Leukotriene antagonist vs. antihistamine

Four studies provided head-to-head data comparing LTRA *vs.* antihistamines [13,19,22,23]. The study by Di Lorenzo et al. [13] included this comparison, and showed that desloratadine had significantly greater efficacy compared to montelukast with regard to TSS (mean difference 1.66, 95% CI 1.28- -1.05), pruritus (mean difference 0.7, 95% CI-0.74- -0.66), number of hives (mean difference −0.21 95% CI −0.27- -0.16), size of largest hives (mean difference −0.24 95% CI −0.29- -0.19) on reflective evaluation. Similarly, on instantaneous evaluation of interference with sleep and the use of rescue medication, desloratadine was shown to be superior to montelukast, although this benefit was not demonstrated with regards to interference with daily activities.

Nettis et al. [23] compared montelukast with fexofenadine in patients with CIU in a double blind study involving 15 patients in montelukast and 12 patients in fexofenadine group. The total symptom score was worse in the fexofenadine group compared with the montelukast group. However the reduction in time of symptomatic profile was the same in both groups.

Godse [22] compared montelukast with cetrizine in patients with CIU, in a randomised trial of 20 patients. The outcome measure was improvement in UAS and severity of itching at one week and two weeks after commencing therapy. Results with montelukast were disappointing, with 8 of the 10 patients in the montelukast group reporting worsening of itching and an increase in the UAS; 2 patients were lost to follow-up. The study had to be terminated at 7 days as a result of patients demanding rescue medication (i.e., cetrizine).

## Conclusions

Our systematic review of randomized controlled trials did not demonstrate strong clinical evidence to refute or accept the practice of prescribing LTRA in patients with chronic urticaria. Reduction in number of hives was seen with LTRAs compared to placebo, although LTRA did not show benefit for any of the other outcomes. Thus, the use of LTRA alone for chronic urticaria is difficult to justify based on current evidence.

Combined therapy of antihistamine with LTRA seems to be beneficial according to most of the studies [14,20,21] although one study gives contradictory results [13]. Head to head comparison of antihistamine versus leukotriene antagonists also gives conflicting data, and no firm conclusion can be made; overall it appears that antihistamines are superior to LTRA. However there is very limited data on this.

The most important difficulty in comparing outcome between the different groups was the wide variability of outcome measures assessed. Even the cumulative scores used to assess overall outcome had wide variation, which is clearly shown in the study by Kosnik et al. [15] where different results were obtained with the two in-house scoring systems compared with the validated UAS. As a result, data pooling for the purpose of meta-analysis was not possible. We attempted to obtain raw data by contacting the authors, but were not successful despite repeated attempts. Another limitation of our review is that because of the wide heterogeneity in study populations, it is difficult to ascertain if LTRA would be more beneficial in patients with more severe, or refractory, chronic urticaria.

Overall, current evidence supports the use of LTRA in combination with antihistamines, and the add-on effect of LTRA in patients currently on antihistamines is likely to be beneficial; this is in agreement with the 2009 treatment guidelines [7]. LTRA cannot, however, be recommended as single therapy for chronic urticaria. LTRA are also significantly more costly, with one month’s treatment with montelukast and zafirlukast costing about GBP 30 and 20 respectively while cetirizine costs just GBP 0.85 and desloratadine costs GBP 7 for the same duration [24].

Importantly, LTRA appeared well tolerated with low side effect profiles; this was demonstrated repeatedly in nearly all trials. Thus, LTRA can be safely recommended in combination with antihistamines in patients with chronic urticaria who show inadequate response to antihistamines alone.

There is clearly a need for further studies. Future randomized controlled studies should stratify patients based on severity of chronic urticaria using standardized criteria. Outcome measures should be carefully chosen, using accepted and validated scales of measurement. Effects on quality of life are an important outcome measure to be considered. Studies should also be powered sufficiently to evaluate the effect of benefit in subgroups of patients with ASST positivity, food allergy, acetylsalicylic acid and NSAID hypersensitivity. However this may not always be feasible, thus alternately studies should use stringent inclusion criteria to define the type of urticaria clearly, in order to make comparisons and recommendations clearly.

## Competing interests

The authors declared that they no competing interest.

## Authors’ contributions

SR and NLDS suggested the idea for this review. NLDS, HD, and CR searched the literature and identified eligible studies for inclusion; ACR and SR independently validated these. NLDS, HD, CR and SR performed the meta analysis; NLDS and SR wrote the first draft. CR, ACR and SR critically reviewed the manuscript. All authors read and confirmed the final manuscript.
